# Editorial: Challenges for Obesity in the 21st Century: Psychology, Nutrition, Modern Lifestyle Behavior and Neuroendocrine Responses

**DOI:** 10.3389/fnut.2022.887272

**Published:** 2022-04-12

**Authors:** Danielle Arisa Caranti, Daniela Sayuri Inoue, Fabio Santos Lira

**Affiliations:** ^1^Post Graduate Program of Interdisciplinary Health Sciences, Federal University of São Paulo, São Paulo, Brazil; ^2^Obesity Study Group (GEO), Post-graduate Program of Nutrition, Federal University of São Paulo, São Paulo, Brazil; ^3^Department of Health, Education and Society, Federal University of São Paulo, São Paulo, Brazil; ^4^Department of Biosciences, Federal University of São Paulo, São Paulo, Brazil; ^5^Exercise and Immunometabolism Research Group, Post-graduation Program in Movement Sciences, Department of Physical Education, Universidade Estadual Paulista (UNESP), São Paulo, Brazil

**Keywords:** cognitive nutrition, lifelong healthy behavior, interdisciplinary treatment strategies, obesity, exercise

## Introduction

Obesity is a worldwide health problem with pandemic proportions. Although its multi-factorial etiology is already recognized, psychological aspects are increasingly gaining attention in studies related to obesity. The serious fundamental question in the modern pandemic of obesity relates to the choices individuals make in an obesogenic environment. Diverse modern cultural features and personal lifestyles interact with the vulnerable metabolic physiology of these individuals. Depression, anxiety, and stress are also closely linked to obesity. Genetic, epigenetic, neuroendocrinal, and immunometabolic changes play a pivotal role in understanding the pathophysiological condition and the psychology of these individuals. Lack of comprehensive knowledge on obesity-related issues makes both prevention and treatment difficult.

The present Research Topic considered the psychobiological and neuroendocrine mechanisms of appetite, exercise, cognitive behavior, lifestyle habits, and food intake, and whether these factors, in terms of obesity prevention and treatment, will prove to be relevant in clinical practice and behavioral strategies. Thus, understanding the dimension of the interaction between biological, psychological, and environmental domains for the emergence and maintenance of obesity will likewise assist in combating this disease.

The schedule was organized with specific sub-topics of interest including (1) Increase the visibility of causes and consequences of our modern lifestyle resulting in obesity, (2) Food intake cross-talk between emotions and neuroendocrine control: the role of exercise, (3) Lifelong healthy behavior and psychoneuroimmunology aspects, (4) Cognitive nutrition and future treatments in obesity, (5) Potential interdisciplinary treatment for obesity and the use of Cognitive and Behavioral Therapy, and finally (6) Experimental models linked to nutrition, behavior and neuroendocrine mechanisms.

Although studies related to obesity intensified decades ago, ranging from elucidations of pathophysiological mechanisms to different types of pharmacological and non-pharmacological treatments, such as multidisciplinary treatments, it is necessary to admit that the advancement of science and technology, wider knowledge, and critical reflection on such these fields have allowed studies of obesity go through increasingly segmented and refined levels. In this Research Topic, we bring to you four studies that shed light on some of the consequences of contemporary lifestyle impacting obesity control in the twenty-first century.

“*Does Modern Lifestyle Favor Neuroimmunometabolic Changes? A Path to Obesity*” is a review article elaborated by Marques et al. in which they discuss the problematization of obesity in a very fluid way, addressing the global trend toward a lifestyle characterized by a positive energy balance. More palatable and ultra-processed foods, technological advances and greater urbanization, reduced sleep time as a result of an altered emotional state seem to be part of the global reality for this century. Thus, the authors discuss the relationship between the triad formed by the Western diet, sleep deprivation, and sedentary behavior, which directly influences the positive energy balance and biochemical imbalances that lead to the meta-inflammation present in obesity.

Metainflammation may bring serious changes in adolescents' development when obesity is already installed. Although there are many studies investigating the impact of obesity on the health of adolescents, the original article by Simoes et al. “*Sex-Dependent Dyslipidemia and Neuro-Humoral Alterations Leading to Further Cardiovascular Risk in Juvenile Obesity*,” provides evidence that obesity can lead to different damages according to the sex of the adolescent, since it is a period of human development when endocrine events occur in a heterogeneous way. Simoes et al. present data on the lipid profile in addition to neuropeptides related to the food intake of these adolescents, which potentiate the early onset of cardiovascular damages.

Lifestyle changes are essential for obese individuals to lose weight and maintain a healthy amount of body mass. Research involving multidisciplinary treatment focus precisely to encourage such changes to a healthier lifestyle. The present Research Topic provided two studies that encompass a multidisciplinary intervention.

The original article written by Tamini et al., “*Effects of a 3-Week In-Hospital Multidisciplinary Body Weight Reduction Program in Obese Females: Is Measured Resting Energy Expenditure Essential for Tailoring Adequately the Amount of Energy Intake?*” compared two methods of measuring resting metabolic rate (RMR) and elucidated how the estimation of resting energy expenditure is essential for an accurate prescription of dietary calories in a multidisciplinary weight loss program. A false estimate of the RMR can make it significantly difficult to reduce body mass.

The multidisciplinary study developed by Moraes et al. “*Cognitive Behavioral Approach to Treat Obesity: A Randomized Clinical Trial*” highlights the advantages of the cognitive-behavioral approach in the multidisciplinary treatment of obesity when compared to an approach involving education and health programs and an approach including a physical exercise program. This study reinforces the importance of intergrading different areas of health to effectively combat this epidemic.

All the articles found and revealed behavior modification techniques, including behavior interventions to reduce obesity and a human context about the quality of life including an approach in the multidisciplinary treatment of obesity when compared to an approach involving education and health programs or a traditional model using a disciplinary therapy by exercise.

In this way, behavior strategies including interdisciplinary therapy and social support are necessary to treat obesity and comorbidities. Behavior modification includes goal-setting, stimulus control, stress factors management, self-monitoring, cognitive restructuring, stress management, problem-solving, and support systems. Behavioral interventions, exercise, and diet lead to more effective and sustainable weight maintenance. Health care providers must become more involved in preventing obesity and incorporate multiple behavioral interventions in managing and preventing obesity. There is a need to transition from prescribing only diet and exercise as lifestyle interventions to a more holistic approach that includes behavioral treatment (1).

The state of the art of this topic is a challenge to new strategies about the modern behavior lifestyle. We are collaborating to find a new direction when we are promoting a better quality of life and an interdisciplinary way to treat obesity, we are reinforcing the approach using a behavioral strategy to care for a complex disease in this Research Topic.

## Author Contributions

FL, DI, and DC participated all process. All authors contributed to the article and approved the submitted version.

## Conflict of Interest

The authors declare that the research was conducted in the absence of any commercial or financial relationships that could be construed as a potential conflict of interest.

## Publisher's Note

All claims expressed in this article are solely those of the authors and do not necessarily represent those of their affiliated organizations, or those of the publisher, the editors and the reviewers. Any product that may be evaluated in this article, or claim that may be made by its manufacturer, is not guaranteed or endorsed by the publisher.
